# Multi-centric AI Model for Unruptured Intracranial Aneurysm Detection and Volumetric Segmentation in 3D TOF-MRI

**DOI:** 10.1007/s10278-025-01533-3

**Published:** 2025-05-12

**Authors:** Ashraya Kumar Indrakanti, Jakob Wasserthal, Martin Segeroth, Shan Yang, Andrew Phillip Nicoli, Victor Schulze-Zachau, Johanna Lieb, Joshy Cyriac, Michael Bach, Marios Psychogios, Matthias Anthony Mutke

**Affiliations:** 1 Department of Diagnostic and Interventional Neuroradiology, Basel University Hospital, Petersgraben 4, 4031 Basel, Switzerland; 2 Clinic of Radiology and Nuclear Medicine, University Hospital Basel, Petersgraben 4, 4031 Basel, Switzerland

**Keywords:** Intracranial aneurysm detection, Intracranial aneurysm segmentation, 3D TOF-MRI, nnU-Net

## Abstract

The aim of this study was to develop an open-source nnU-Net-based AI model for combined detection and segmentation of unruptured intracranial aneurysms (UICA) in 3D TOF-MRI and compare models trained on datasets with aneurysm-like differential diagnoses. This retrospective study (2020–2023) included 385 anonymized 3D TOF-MRI images from 345 patients (mean age 59 years, 60% female) at multiple centers plus 113 subjects from the ADAM challenge. Images featured untreated or possible UICA and differential diagnoses. Four distinct training datasets were created, and the nnU-Net framework was used for model development. Performance was assessed on a separate test set using sensitivity and false positive (FP)/case rate for detection and DICE score and NSD (normalized surface distance, 0.5 mm threshold) for segmentation. Segmentation performance on the test set was also compared to a second human reader. The four models achieved overall sensitivity between 82 and 85% and an FP/case rate of 0.20 to 0.31, with no significant differences (*p* = 0.90 and *p* = 0.16) between them. The primary model showed 85% sensitivity and 0.23 FP/case rate, outperforming the ADAM-challenge winner (61%) and a nnU-Net trained on ADAM data (51%) in sensitivity (*p* < 0.05). Mean DICE (0.73) and NSD (0.84 for 0.5 mm threshold) for correctly detected UICA did not significantly differ from human reader performance. Our open-source, nnU-Net-based AI model (available at https://zenodo.org/records/13386859) demonstrates high sensitivity, low FP rates, and consistent segmentation accuracy for UICA detection and segmentation in 3D TOF-MRI, suggesting its potential to improve clinical diagnosis and monitoring of UICA.

**Supplementary Information:**

The online version contains supplementary material available at 10.1007/s10278-025-01533-3.

## Introduction

Unruptured intracranial aneurysms (UICA) affect approximately 3% of the population and pose a significant risk for subarachnoid hemorrhage upon rupture, associated with high morbidity and mortality rates [1, 2]. Early detection and precise measurement of UICA size are critical for effective monitoring and potential treatment [3] to prevent such debilitating outcomes. For UICA assessment, 3D TOF-MRI is most used, primarily due to its noninvasiveness, absence of radiation, and lack of contrast agents [3]. Many UICA are detected incidentally during routine clinical imaging for unrelated pathologies. Additionally, follow-up imaging to monitor UICA for prognostically relevant changes in size and shape is also usually conducted with 3D TOF-MRI. Detecting and segmenting small UICA in these images is challenging, with radiologists’ sensitivity estimated between 60 and 85% [4, 5]. However, comprehensive studies evaluating human error rates in routine imaging are lacking.

Recent advances in artificial intelligence (AI) have the potential to transform radiology [6]. Examples are lung nodule detection on chest radiographs [7], skin cancer classification in dermatology [8], and AI-enhanced ECG usage in cardiology [9]. Regarding detection and segmentation tasks in medical imaging, the nnU-Net, a particular type of AI system, has shown significant potential due to its self-configuring capabilities [10].

While multiple studies have explored various algorithms for UICA detection [11–14], few have focused on a single model capable of detection and segmentation [15, 16]. Moreover, we incorporate challenging and potentially confounding differential diagnoses, such as infundibula and vascular loops, in the training process. These are crucial for clinical accuracy, and their omission from training datasets might result in reduced sensitivity in clinical scenarios.

This study aims to develop an AI model for combined detection and 3D segmentation of UICA in 3D TOF-MRI brain scans and to compare the performance of models trained on additional datasets containing potential confounding diagnoses. The primary objective was to assess the performance of the models in accurately detecting and segmenting confirmed UICA. We compared their performance to the performance of the ADAM-challenge winner [17] and to an nnU-Net model trained solely on the ADAM-challenge dataset. The secondary objective was to compare the performances of these differently trained models and ascertain if significant variations existed among them. Such a model could enhance the accuracy of UICA detection and segmentation, ultimately reducing missed and false diagnoses, and aiding in the monitoring and therapy decisions for UICA.

## Materials and Methods

### Data Acquisition

This retrospective study received an ethics waiver from the local institutional Review Board under project-ID Req-2024–00337.

**Fig. 1:**
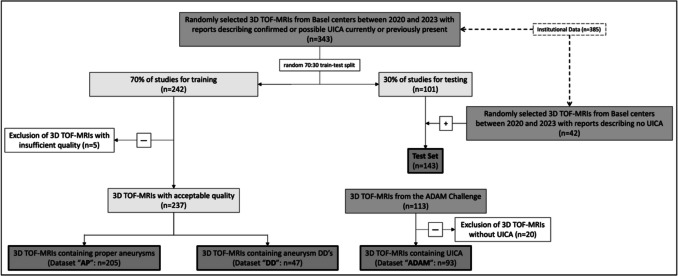
Study flowchart. After collection of 3D TOF-MRIs possibly containing UICA and excluding images not fulfilling inclusion criteria or with insufficient quality, the data was reorganized into four distinct datasets: AP, DD, ADAM, and test datasets. The first three datasets were used for model training in different combinations (AP, AP + DD, AP + ADAM, AP + DD + ADAM), while the test dataset was used for model performance evaluation and model comparison

We performed a retrospective, diagnostic imaging study using 385 randomly sampled, anonymized, non-contrast 3D TOF-MRI images acquired from 345 patients between 2020 and 2023 in a clinical imaging setting from our institute and its affiliates (Institutional Data) (see Fig. 1 for a comprehensive data flowchart). Of the 385 studies, 343 studies contained at least one confirmed or possible UICA (i.e., positives), and the remaining 42 studies contained no UICA (i.e., negatives). Additionally, we used the dataset from the ADAM challenge [17]. Further acquisition details are provided in Supplementary Table 1.

Inclusion criteria were studies containing one or more untreated UICA. Also, studies with UICA differential diagnoses (infundibula, vascular loops, fenestrations, and focal ectasias) were included, i.e., the uncertainty of the radiologist prevented a definitive diagnosis between a true aneurysm or one of the differential diagnoses listed above. The positives data was then split into training (70%, *n* = 242) and test data (30%, *n* = 101). The only exclusion criterium for studies was the lack of sufficient imaging quality of the TOF-MRIs, resulting in the dataset containing diverse, non-aneurysmatic cerebral pathologies. The negatives data was added to the test data.

The mean age of Institutional Data subjects was 59 years (range, 5–88 years), 60% being female (female ages 9–87 years, mean 60 years; male ages 5–88 years, mean 57 years). For the ADAM data, the median age was 55 years (range, 24 years to 75 years) and 75% female [17].

### Data Categorization

We categorized the training data into three distinct training datasets for comprehensive analysis (Fig. 1). The first dataset “Aneurysm Proper” (AP, *n* = 205) included only studies where an UICA was diagnosed in the clinical reports. The second dataset “Aneurysm Differential Diagnoses” (DD, *n* = 47) included studies where a UICA differential diagnosis was identified in the routine clinical reports. To introduce institutional variability, 93 out of 113 studies from the ADAM challenge (all positive studies containing at least one UICA) yielded the third “ADAM” dataset. For the final test dataset, in addition to the 101 test studies containing only UICA and no UICA differential diagnoses, 42 randomly sampled 3D TOF-MRI images acquired between 2020 and 2023 with no reported UICA were added.

### Data Segmentation

The voxel-by-voxel segmentation of the Institutional Data studies (training and test sets) was performed by a junior medical doctor, guided by the corresponding radiology reports. This task was supervised by two board-certified neuroradiologists (with 10 and 15 years of experience). In instances where the segmentation posed challenges, disagreements or deviated from initial radiology reports, a consensus was established among all parties involved. Additionally, the test set was segmented by a second junior medical doctor, also guided by the radiology reports and supervised by a board-certified neuroradiologist. This second reader is referred to as the human reader, while the segmentations of the first reader are considered the ground truth. The ADAM-challenge dataset is pre-segmented.

In the AP dataset, all proper UICA were segmented. In the DD dataset, all UICA differential diagnoses were segmented. The manual segmentation and refinement of the preliminary segmentations were performed using the medical image editing software NORA [18].

### Model Training and Evaluation

We utilized the nnU-Net framework [10] to develop a model for detection and segmentation of UICA. The nnU-Net is a U-Net based framework designed for medical segmentation which automatically optimizes both data preprocessing and U-Net hyperparameter selection in a standardized manner. It determines the best settings for data preprocessing steps like resampling and normalization and for U-Net architectural choices such as layer count and batch size, by analyzing dataset characteristics and applying heuristics on these. It also applies a wide range of data augmentation techniques internally (including rotations, mirroring, blurring, scaling). Over the last years, this framework has proven to be very effective in various segmentation applications [19–22].

The nnU-Net’s self-configuring pipeline performs key preprocessing steps (intensity normalization, adaptive resampling; see the Supplementary Notes of [10], for a full list), and we did not apply any additional preprocessing. In preliminary experiments, N4 bias-field correction yielded no measurable gains in sensitivity, FP/case, or segmentation accuracy. By relying solely on nnU-Net’s standardized defaults, we aimed to preserve model generalizability and avoid potential overfitting from ad-hoc image preprocessing.

Testing was performed on the separate test dataset described above. UICA were considered detected if there was any overlap (> 0%) between the prediction and the ground truth lesion. Detection metrics included sensitivity and false positive (FP)/case (the average number of false positive results per imaging study). The performance of the segmentations was evaluated using the lesion-wise DICE score and lesion-wise normalized surface distance (NSD) with 0.5 mm threshold for all correctly detected UICA. This approach was used to simulate clinical practice, where a radiologist would only assess correctly detected UICA for segmentation analogous to [17]. We extracted maximal diameter and volume of the UICA. Using these metrics, the IH models were compared amongst each other, and compared to the open-source winning model of the ADAM challenge [23]; henceforth referred to as the ADAM-winner model) and to a simple, standard nnU-Net model trained only on the ADAM dataset using default settings, henceforth called the ADAM-nnU-Net model.

### Statistical Analysis

Detection was evaluated using the χ^2^-test. Mann–Whitney *U* and Kruskal–Wallis tests were used to compare lesion sizes and segmentation accuracy on the test set. 95% CI were calculated via nonparametric percentile Bootstrapping (*N* = 10^4^) and are reported in square brackets after metric values. *p*-values < 0.05 were considered statistically significant. Hypothesis testing was two-tailed. Statistical analysis was performed in Python (version 3.11) with SciPy (version 1.11.3) [24].

## Results

### Data

All UICA in the Institutional Data were saccular, with very few exceptions: seven were fusiform and one was mycotic. Since the only exclusion criterium for studies was insufficient imaging quality, the dataset also encapsulated a wide range of non-vascular pathologies.

The study included four datasets: AP (237 studies, 270 lesions), DD (47 studies, 54 lesions), ADAM (93 studies, 125 lesions), and the test set (142 studies, 124 lesions). The maximum aneurysm diameters (mean ± SD, median) were 4.37 ± 2.73 mm, 3.78 mm (AP); 2.4 ± 1.01 mm, 2.41 mm (DD); 4.53 ± 2.19 mm, 4.34 mm (ADAM); and 4.45 ± 3.49 mm, 3.58 mm (test set).

Aneurysm volumes (mean ± SD, median) were 52.83 ± 155.03 mm^3^, 14.71 mm^3^ (AP); 5.23 ± 4.14 mm^3^, 3.94 mm^3^ (DD); 31.76 ± 48.66 mm^3^, 16.35 mm^3^ (ADAM); and 95.07 ± 388.65 mm^3^, 13.02 mm^3 ^(test set).

### Performance of AP + DD + ADAM Model

The AP + DD + ADAM model, which is the primary and largest model of our study, demonstrated a sensitivity of 0.85 [0.78, 0.91] and an FP/case rate of 0.23 [0.16, 0.30] for UICA. For the correctly detected UICA, it showed a high lesion-wise DICE score of 0.73 [0.69, 0.77] (measuring overlap accuracy across individual UICA).

**Fig. 2:**
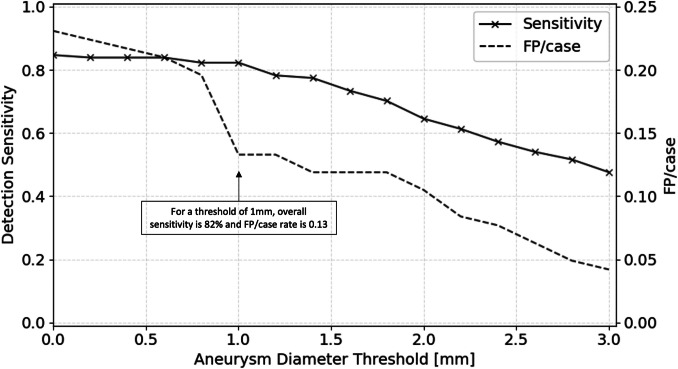
Threshold-dependent sensitivity and FP/case rate of the AP + DD + ADAM model. While both metrics decrease with increasing aneurysm diameter thresholds, the FP/case rate drops more rapidly, showing a pronounced decline at a threshold of 1 mm

In a thresholding post-processing [16], predictions smaller than a defined size threshold were discarded, and only those exceeding the threshold were retained. Thresholding led to a marked reduction in the FP/case rate, while maintaining a comparably high sensitivity. As shown in Fig. 2, applying a size threshold of 1 mm resulted in a steep decline in FP/case rate, whereas sensitivity remained high at 0.82.

FP predictions of the model were reviewed by a board-certified neuroradiologist post-hoc. All were within blood vessels, and none was extravascular. 48.5% were potentially relevant vascular lesions. These included suspected aneurysms, prominent infundibula, or other ambiguous vascular findings that appeared unusual enough to potentially warrant further investigation in clinical routine. 24.2% were located in venous structures, and 24.2% were due to arterial TOF flow artifacts. One FP was located in an artery at a severely atherosclerotic segment.

Furthermore, for any given correctly detected UICA, about 84% of the predicted segmentation surface remained within a margin of 0.5 mm to the ground truth (lesion-wise NSD = 0.84 [0.80, 0.88]). Size difference between predictions and ground truth was on average 1.21 mm [0.89, 1.59] (maximum diameter) and 59.52 mm^3^ [10.89, 122.42] (volume), respectively.

### Comparison of AP + DD + ADAM Model with Non-IH models

For UICA detection sensitivity, the AP + DD + ADAM model significantly outperformed the ADAM-winner (sensitivity, 0.61 [0.53, 0.70]; *p* < 0.05) and the ADAM-nnU-Net (sensitivity, 0.51 [0.42, 0.60]; *p* < 0.05). There was no significant difference for the total FP/case despite the difference in sensitivity (ADAM-winner, 0.23 [0.16, 0.30]; ADAM-nnU-Net, 0.21 [0.14, 0.28]; *p* = 0.88).

Regarding segmentation performance, there were no significant differences in DICE (0.73 [0.69, 0.77] vs 0.71 [0.66, 0.75], *p* = 0.19) or NSD (0.84 [0.80, 0.88] vs 0.85 [0.81, 0.89], *p* = 0.79) between the AP + DD + ADAM and the ADAM-winner models. However, the AP + DD + ADAM model performed significantly better than the ADAM-nnU-Net in both DICE (0.73 [0.69, 0.77] vs 0.61 [0.54, 0.67], *p* < 0.05) and NSD (0.84 [0.80, 0.88] vs 0.74 [0.68, 0.80], *p* < 0.05).

The AP + DD + ADAM model and the ADAM-winner model reported no significantly different values regarding mean volume differences of the aneurysms (59.52 mm^3^ [10.89, 122.42] vs 78.62 mm^3^ [19.45, 153.39], *p* = 0.68) and mean maximum diameter differences (1.21 mm [0.89, 1.59] vs 2.08 mm [1.49, 2.79], *p* = 0.06) between predictions and ground truth. The AP + DD + ADAM model significantly outperformed the ADAM-nnU-Net in both predicted mean volume differences (59.52 mm^3^ [10.89, 122.42] vs 83.07 mm^3^ [24.49, 159.87], *p* < 0.05) and predicted mean maximum diameter differences (1.21 mm [0.89, 1.59] vs 2.63 mm [2.05, 3.32], *p* < 0.05) between predictions and ground truth.

### Detection Performance of IH Models

**Fig. 3:**
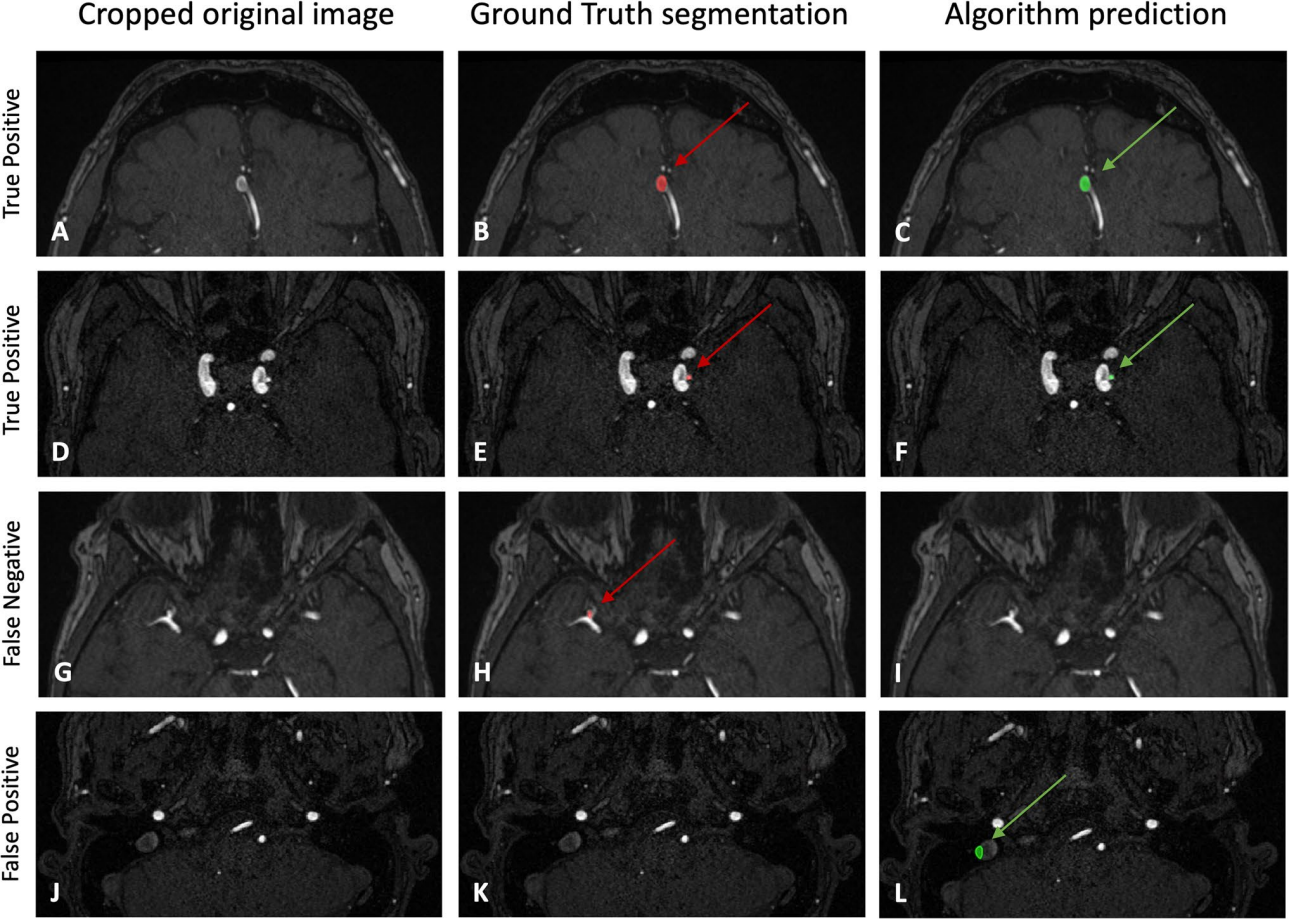
Examples of images, ground truth segmentations and the model predictions (AP + DD + ADAM model). **A**–**C** and **D**–**F** Two examples of correctly identified aneurysms with corresponding segmentations. **G**–**I** Aneurysm missed by model. **J**–**L** FP prediction in the internal jugular vein with no corresponding ground truth aneurysm

Detection and segmentation performance metrics of the models are summarized in Table 1. Figure 3 shows image examples from selected patients.

**Table 1:** Summary of the different models’ performance in both detection and segmentation on the test set. Size differences are calculated as ground truth size − prediction size. The AP + DD + ADAM model is our primary and best performing model (last column), representing the largest and most diverse dataset. All numbers are given as mean with 95% CI. DICE, NSD, volume differences, maximum diameter differences, and their Spearman correlation coefficients are given for correctly detected aneurysms

	AP	AP **+ **DD	AP **+ **ADAM	ADAM**-**nnU**-**Net	ADAM-winner	AP **+ **DD **+ **ADAM
**Sensitivity** Across all aneurysms aneurysms < 2 mm aneurysms < 4 mm aneurysms ≥ 4 mm	0.82 [0.76,0.89] 0.40 [0.19,0.61] 0.73 [0.62,0.83] 0.94 [0.88,1.00]	0.85 [0.78,0.91] 0.45 [0.23,0.67] 0.76 [0.66,0.86] 0.96 [0.91,1.00]	0.83 [0.76,0.90] 0.45 [0.23,0.67] 0.71 [0.61,0.82] 0.98 [0.95,1.00]	0.51 [0.42, 0.60] 0.15 [0.00, 0.31] 0.36 [0.24, 0.47] 0.70 [0.58, 0.83]	0.61 [0.53, 0.70] 0.15 [0.00, 0.31] 0.50 [0.38, 0.62] 0.76 [0.65, 0.87]	0.85 [0.78,0.91] 0.35 [0.14,0.56] 0.74 [0.64,0.85] 0.98 [0.95,1.00]
**FP/case** Across all aneurysms aneurysms < 2 mm aneurysms < 4 mm aneurysms ≥ 4 mm	0.20 [0.13, 0.26] 0.13 [0.08, 0.19] 0.18 [0.12, 0.25] 0.01 [0, 0.03]	0.31 [0.23, 0.38] 0.21 [0.14, 0.28] 0.30 [0.23, 0.38] 0.01 [0, 0.02]	0.24 [0.17, 0.31] 0.16 [0.10, 0.22] 0.21 [0.14, 0.28] 0.03 [0, 0.05]	0.21 [0.14, 0.28] 0.13 [0.07, 0.18] 0.20 [0.14, 0.27] 0.01 [0, 0.02]	0.23 [0.16, 0.3] 0.13 [0.07, 0.18] 0.21 [0.14, 0.28] 0.02 [0, 0.04]	0.23 [0.16, 0.3] 0.13 [0.07, 0.18] 0.22 [0.15, 0.28] 0.01 [0, 0.03]
**DICE**	0.67 [0.62, 0.71]	0.7 [0.66, 0.74]	0.68 [0.63, 0.72]	0.61 [0.54, 0.67]	0.71 [0.66, 0.75]	0.73 [0.69, 0.77]
**NSD (0.5 mm)**	0.81 [0.76, 0.85]	0.84 [0.80, 0.88]	0.81 [0.77, 0.85]	0.74 [0.68, 0.80]	0.85 [0.81, 0.89]	0.84 [0.80, 0.88]
**Volume Differences** [mm^3^]	61.86 [13.51, 124.49]	60.80 [12.49, 122.89]	52.04 [9.67, 109.15]	83.07 [24.49, 159.87]	78.62 [19.45, 153.39]	59.52 [10.89, 122.42]
**Maximum Diameter Differences** [mm]	1.35 [1.04, 1.72]	1.26 [0.95, 1.63]	1.20 [0.95, 1.50]	2.63 [2.05, 3.32]	2.08 [1.49, 2.79]	1.21 [0.89, 1.59]
**Spearman Correlation Coefficient for Volumes**	0.88 [0.82, 0.91]	0.90 [0.86, 0.93]	0.94 [0.91, 0.96]	0.71 [0.56, 0.82]	0.91 [0.86, 0.94]	0.92 [0.88, 0.94]
**Spearman Correlation Coefficient for Maximum Diameters**	0.87 [0.81, 0.91]	0.88 [0.82, 0.91]	0.90 [0.86, 0.93]	0.71 [0.56, 0.81]	0.90 [0.84, 0.93]	0.89 [0.85, 0.93]

IH models achieved an overall sensitivity ranging between 0.82 and 0.85 (no significant difference; *p* = 0.94). Total FP/case rates of the IH models ranged from 0.20 to 0.31 (no significant difference; *p* = 0.16). Ten aneurysms and eight aneurysm DD, which were not described in the clinical reports, were identified by the models and confirmed by the supervising neuroradiologists. These were then included as aneurysms in the datasets.

**Fig. 4:**
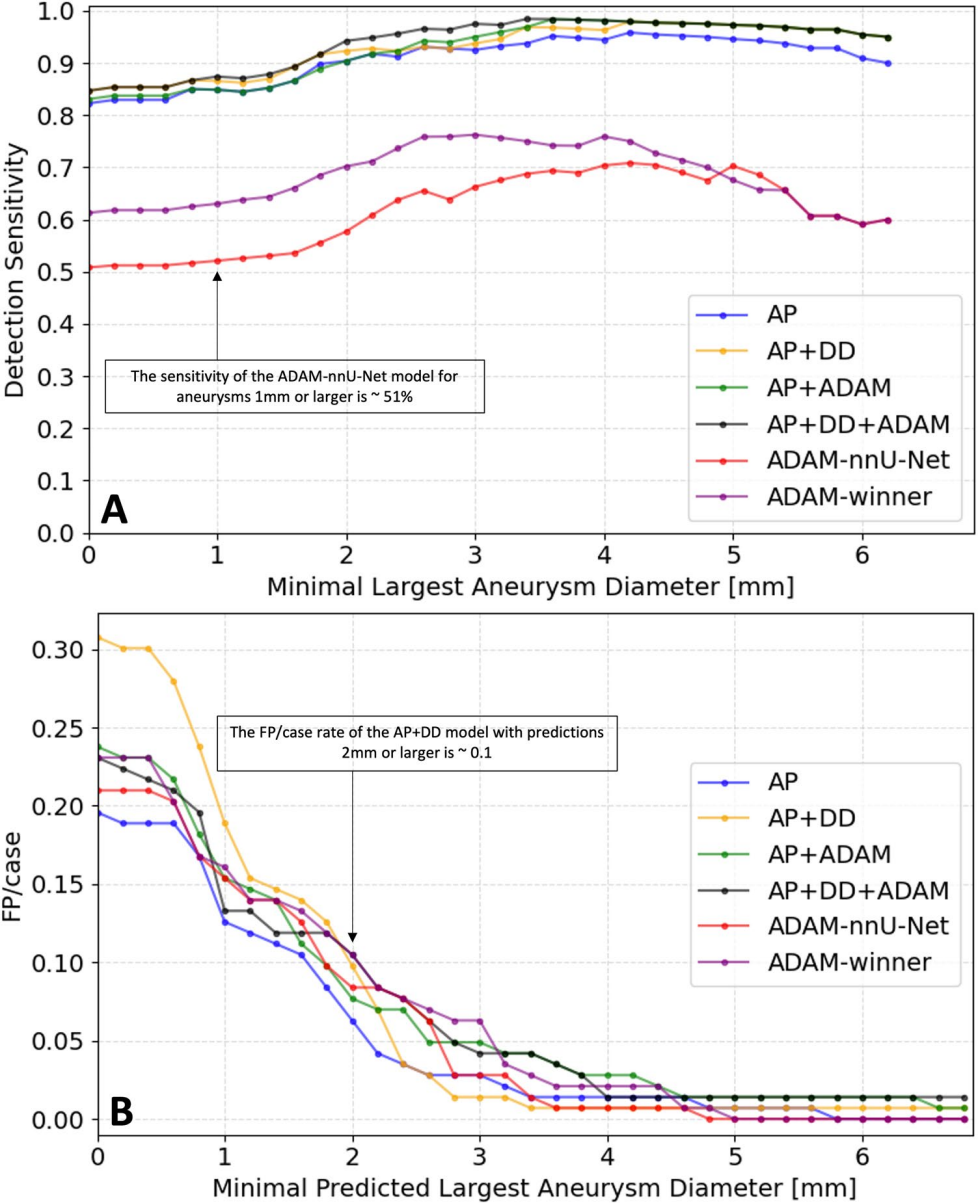
Diameter-dependent cumulative sensitivity (**A**) and FP/case rate (**B**) of all models. The figure shows detection sensitivity and FP/case for all detected aneurysms that are equal or larger than the diameter specified on the *x*-axis

For IH models, sensitivity improved with increasing lesion diameter (Fig. 4a), reaching 0.98 [0.95, 1.00] for UICA > 4 mm for the AP + DD + ADAM model (vs 0.74 [0.64, 0.85] for UICA < 4 mm, *p* < 0.05). Correspondingly, the FP/case rate showed a marked decrease with increasing lesion diameter, with 0.01 [0, 0.03] for predictions > 4 mm (vs 0.22 [0.15, 0.28] for predictions < 4 mm, *p* < 0.05) (Fig. 4b).

### Segmentation Performance of IH Models and Human Readers for Detected Aneurysms

Mean lesion-wise DICE score showed no significant differences across IH models, varying between 0.67 and 0.73 (*p* = 0.10). DICE score showed almost no dependency on lesion size (for a visual representation, refer to Supplementary Fig. 1). Mean lesion-wise NSD varied between 0.81 and 0.84 and showed no significant differences between IH models (*p* = 0.33), meaning that for a given segmentation, between 81 and 84% remained in a margin of 0.5 mm to the ground truth.

Mean lesion-wise DICE of the human reader was 0.74 [0.72, 0.76], and mean lesion-wise NSD of the human reader was 0.89 [0.88, 0.91]. There were no significant differences in DICE (*p* = 0.16) and NSD (*p* = 0.10) between the human reader and the IH models.

### Ground Truth and Predicted Size Comparison

Comparison of the ground truth and predicted UICA sizes showed that for all IH models, the average volume of the predicted lesions was significantly smaller than the average volume of the ground truths. Spearman correlation coefficients between true and predicted sizes were between 0.88 and 0.94 for volumes and 0.87 and 0.90 for largest diameters and did not differ significantly (*p* = 0.07, *p* = 0.61). The average differences between ground truth and model predictions are listed in Table 1. Mean volume differences were between 52 and 61 mm^3^ for all IH models and mean maximum diameter differences were between 1.21 and 1.35 mm. There were no significant differences between the IH models for volume (*p* = 0.75) and maximum diameter differences (*p* = 0.73).

## Discussion

In our study, we introduce a deep-learning model based on the nnU-Net to address two major challenges in diagnosing UICA: combined detection and volumetric segmentation on non-contrast 3D TOF-MRIs.

The first challenge, detecting UICA in a routine clinical setting, is difficult due to increasing neuroradiologic workload and time constraints [25]. Many UICA are found during unrelated imaging, where focusing the search pattern on small vessels requires considerable mental effort.

The primary model identified secondary aneurysms missed in initial readings, reducing satisfaction of search errors [25] and improving detection of these not-so-rare cases (Rinne et al., 1994). Furthermore, almost half of the FP lesions predicted by the model were classified as potentially relevant vascular lesions upon review. While classified as FP in our study, these would still be relevant and useful model outputs during clinical routine because they might require further review or follow-up imaging. Excluding these lesions from the FP/case metric would almost half its value to 0.12/case. However, it is important to note that the test set itself does not reflect the actual incidence of UICA in the general population and contains a disproportionally high number of such patients, and it is known that patients with at least one UICA are strongly predisposed to having more such vascular lesions [26]. Therefore, we might expect both the FP/case rate and the fraction of potentially relevant vascular lesions to be lower in clinical routine.

Our primary model trained on multi-center data detected UICA ≥ 4 mm with a high sensitivity of 98% and a low FP/case rate of 1 per 100 cases for findings ≥ 4 mm. This is equal to the capabilities previously reported in humans [27]. UICA < 4 mm are less clinically significant but the model maintained a sensitivity of 74% and a low FP/case rate of 22 per 100 for such small findings. This size-dependent behavior probably mirrors human detection patterns.

However, the metrics listed above do not include thresholding as a post-processing step, as the threshold is a hyperparameter that can be adjusted by the user. By selecting an appropriate detection threshold—such as 1 mm, which appears optimal based on the threshold-dependent performance shown in Fig. 2—an advantageous trade-off can be achieved, maintaining a high sensitivity of 82% while distinctly reducing the FP/case rate to 0.13.

Our metrics exceed previously reported models: [11–13, 28] all conducted multicentric studies for 3D UICA detection with good sensitivity (73%, 89.1%, 93%, 85.7%), but most had higher FP/case rates (0.88/case, 4.2/case, not provided for Ueda et al., 0.09/case). Importantly, none of these studies segmented UICA volumetrically.

We compared different models trained on different dataset combinations, some including clinically relevant and challenging uncertainty with potential differential diagnoses like arterial infundibula [29]. We did not find a significant difference for detection, segmentation, and FP/case rate suggesting a remarkable capability and resilience in complex neurovascular cases.

Our model outperformed the ADAM challenge’s winning model in sensitivity, likely due to our larger, more diverse training dataset with thorough expert segmentation. It also significantly surpassed a basic nnU-Net trained only on the ADAM dataset.

While the ADAM winner model was superior to the basic nnU-Net, probably due to loss function ensembling, it matched our primary model in segmentation quality. However, our model detected significantly more UICA, and those additional detections were segmented with equally high quality.

The second challenge in UICA diagnosis is accurately measuring aneurysm size and volume. Changes in size over time can indicate an increased risk of rupture, potentially necessitating further diagnostic or interventional procedures [3]. Therefore, precise UICA segmentation and volumetry with the extraction of maximal diameter and volume are relevant clinical features.

Considering the small size and complexity of UICA, our primary model demonstrated high accuracy in segmentation: It achieved an average DICE score of 0.73 for all correctly detected UICA. Additionally, a mean normalized surface distance of 0.84 indicates that on average, 84% of the predicted segmentation surface is within one voxel of the ground truth, using a 0.5 mm threshold on 3D TOF-MRI scans with a slice thickness of 0.5 mm. The high NSD value indicates a very reliable segmentation quality when an aneurysm is correctly detected. The model was able to extract maximal diameter and volume of UICA with excellent correlation between ground truth and prediction. The first measure is integrated into the clinically relevant PHASES score [30] and the second offers a more comprehensive assessment of its true size and therefore its potential impact. However, DICE and NSD were not size-dependent: For larger UICA, segmentation quality generally improved, but so did the incidence of UICA with inhomogeneous signal intensities (e.g., due to partial thrombosis or flow turbulences). The models struggled with such low-intensity regions, leading to poorer segmentations.

To contextualize segmentation performance given the small size of the UICA, we conducted a second segmentation of the test set by another human reader. No significant differences were found between the human reader and the IH models in DICE and NSD scores, indicating human-level performance of the models.

So far, only two models combined detection and volumetric segmentation of UICA in non-contrast 3D TOF-MRIs: [16] had a sensitivity of 90% and a very high FP/case rate of 6.1, required extensive preprocessing, and were evaluated with UICA almost double in size compared to our dataset. [15] had a sensitivity of 78% and a FP/case rate of 0.5, both metrics being inferior to our model. Our model could assist in assessing volumetric changes, where human performance is limited [31]. However, this requires further validation.

Our study has some limitations: While developed on multiple scanners with different field strengths and data from multiple imaging centers, it lacks further prospective evaluation in other centers. An important future step would be the performance validation of the model on a dataset which reflects the incidence of UICA in the general population more accurately. Further, we did not directly demonstrate non-inferiority compared to radiologists with regard to detection performance.

## Conclusion

Our open-source nnU-Net-based AI model for UICA detection and segmentation on 3D TOF-MRI achieved 85% sensitivity and a low FP/case rate of 0.23. It also showed strong segmentation performance with a DICE score of 0.73 and an NSD of 0.84 similar to a human reader. This model could improve UICA detection in routine clinical settings and assist in monitoring aneurysm size over time. The model can be accessed open source under https://zenodo.org/records/13386859 for research purposes.

## Supplementary Information

Below is the link to the electronic supplementary material.

Supplementary file1 (DOCX 232 KB)
