# Correction: Multi-centric AI Model for Unruptured Intracranial Aneurysm Detection and Volumetric Segmentation in 3D TOF-MRI

**DOI:** 10.1007/s10278-025-01569-5

**Published:** 2025-06-25

**Authors:** Ashraya Kumar Indrakanti, Jakob Wasserthal, Martin Segeroth, Shan Yang, Andrew Phillip Nicoli, Victor Schulze-Zachau, Johanna Lieb, Joshy Cyriac, Michael Bach, Marios Psychogios, Matthias Anthony Mutke

**Affiliations:** 1 Department of Diagnostic and Interventional Neuroradiology, Basel University Hospital, Petersgraben 4, 4031 Basel, Switzerland; 2 Clinic of Radiology and Nuclear Medicine, University Hospital Basel, Petersgraben 4, 4031 Basel, Switzerland

**Correction: Journal of Imaging Informatics in Medicine**

10.1007/s10278-025-01533-3

Table 1 was presented incorrectly in this paper. The correct version is below:

**Table 1:** Summary of the different models' performance in both detection and segmentation on the test set. Size differences are calculated as ground truth size – prediction size. The AP+DD+ADAM model is our primary and best performing model (grey column), representing the largest and most diverse dataset. All numbers are given as mean with 95%-CI. DICE, NSD, Volume Differences, Maximum Diameter Differences and their Spearman Correlation Coefficient are given for correctly detected aneurysms

	AP	AP+DD	AP+ADAM	ADAM-nnU-Net	ADAM-winner	AP+DD+ADAM
**Sensitivity** Across all aneurysms aneurysms < 2mm aneurysms < 4mm aneurysms ≥ 4mm	0.82 [0.76,0.89] 0.40 [0.19,0.61] 0.73 [0.62,0.83] 0.94 [0.88,1.00]	0.85 [0.78,0.91] 0.45 [0.23,0.67] 0.76 [0.66,0.86] 0.96 [0.91,1.00]	0.83 [0.76,0.90] 0.45 [0.23,0.67] 0.71 [0.61,0.82] 0.98 [0.95,1.00]	0.51 [0.42, 0.60] 0.15 [0.00, 0.31] 0.36 [0.24, 0.47] 0.70 [0.58, 0.83]	0.61 [0.53, 0.70] 0.15 [0.00, 0.31] 0.50 [0.38, 0.62] 0.76 [0.65, 0.87]	0.85 [0.78,0.91] 0.35 [0.14,0.56] 0.74 [0.64,0.85] 0.98 [0.95,1.00]
**FP/case** Across all aneurysms aneurysms < 2mm aneurysms < 4mm aneurysms ≥ 4mm	0.20 [0.13, 0.26] 0.13 [0.08, 0.19] 0.18 [0.12, 0.25] 0.01 [0, 0.03]	0.31 [0.23, 0.38] 0.21 [0.14, 0.28] 0.30 [0.23, 0.38] 0.01 [0, 0.02]	0.24 [0.17, 0.31] 0.16 [0.10, 0.22] 0.21 [0.14, 0.28] 0.03 [0, 0.05]	0.21 [0.14, 0.28] 0.13 [0.07, 0.18] 0.20 [0.14, 0.27] 0.01 [0, 0.02]	0.23 [0.16, 0.3] 0.13 [0.07, 0.18] 0.21 [0.14, 0.28] 0.02 [0, 0.04]	0.23 [0.16, 0.3] 0.13 [0.07, 0.18] 0.22 [0.15, 0.28] 0.01 [0, 0.03]
**DICE**	0.67 [0.62, 0.71]	0.7 [0.66, 0.74]	0.68 [0.63, 0.72]	0.61 [0.54, 0.67]	0.71 [0.66, 0.75]	0.73 [0.69, 0.77]
**NSD (0.5mm)**	0.81 [0.76, 0.85]	0.84 [0.80, 0.88]	0.81 [0.77, 0.85]	0.74 [0.68, 0.80]	0.85 [0.81, 0.89]	0.84 [0.80, 0.88]
**Volume Differences** [mm^3^]	61.86 [13.51, 124.49]	60.80 [12.49, 122.89]	52.04 [9.67, 109.15]	83.07 [24.49, 159.87]	78.62 [19.45, 153.39]	59.52 [10.89, 122.42]
**Maximum Diameter Differences** [mm]	1.35 [1.04, 1.72]	1.26 [0.95, 1.63]	1.20 [0.95, 1.50]	2.63 [2.05, 3.32]	2.08 [1.49, 2.79]	1.21 [0.89, 1.59]
**Spearman Correlation Coefficient for Volumes**	0.88 [0.82, 0.91]	0.90 [0.86, 0.93]	0.94 [0.91, 0.96]	0.71 [0.56, 0.82]	0.91 [0.86, 0.94]	0.92 [0.88, 0.94]
**Spearman Correlation Coefficient for Maximum Diameters**	0.87 [0.81, 0.91]	0.88 [0.82, 0.91]	0.90 [0.86, 0.93]	0.71 [0.56, 0.81]	0.90 [0.84, 0.93]	0.89 [0.85, 0.93]

