# Supplementary material for: Multi-centric AI Model for Unruptured Intracranial Aneurysm Detection and Volumetric Segmentation in 3D TOF-MRI
Source: J Imaging Inform Med. 2025 May 12;39(1):345–54. doi: 10.1007/s10278-025-01533-3 (PMC12920843; doi:10.1007/s10278-025-01533-3)
Supplement: Supplementary file 1 — Supplementary file1 (DOCX 232 KB) [file 10278_2025_1533_MOESM1_ESM.docx]

Supplementary Files

Tables

| Scanner Model | Magnetic Field Strength [T] | T_E_ [ms] | T_R_ [ms] | Slice Thickness [mm] | # of studies |
| --- | --- | --- | --- | --- | --- |
| MAGNETOM Vida | 3.00 | 3.69 | 21.14 | 0.30 | 93 |
| Skyra | 3.00 | 3.42 | 21.00 | 0.50 | 90 |
| Skyra_fit | 3.00 | 3.42 | 21.00 | 0.50 | 75 |
| Avanto_fit | 1.50 | 7.00 | 23.00 | 0.50 | 40 |
| MAGNETOM Sola | 1.50 | 7.15 | 23.32 | 0.47 | 39 |
| Skyra | 3.00 | 3.43 | 21.00 | 0.50 | 15 |
| MAGNETOM Vida fit | 3.00 | 3.69 | 21.00 | 0.40 | 13 |
| Skyra | 3.00 | 3.42 | 19.00 | 0.60 | 5 |
| MAGNETOM Free.Max | 0.55 | 7.07 | 27.67 | 0.51 | 4 |
| MAGNETOM Vida | 3.00 | 3.69 | 21.18 | 0.30 | 3 |
|  |  |  | 21.22 | 0.30 | 2 |
| Skyra | 3.00 | 3.19 | 20.00 | 0.70 | 2 |
| Skyra_fit | 3.00 | 3.42 | 19.00 | 0.60 | 1 |
|  |  |  |  | 0.60 | 1 |
| Skyra | 3.00 | 3.42 | 25.00 | 0.50 | 1 |
| MAGNETOM Sola | 1.50 | 7.00 | 22.40 | 0.60 | 1 |
| MAGNETOM Vida | 3.00 | 3.50 | 20.00 | 0.60 | 1 |
| Prisma | 3.00 | 3.42 | 21.00 | 0.50 | 1 |
| MAGNETOM Vida | 3.00 | 3.69 | 21.00 | 0.40 | 1 |
|  |  | 4.98 | 23.99 | 0.30 | 1 |
|  |  | 3.69 | 21.16 | 0.30 | 1 |
|  |  |  | 21.14 | 0.40 | 1 |
| MAGNETOM Vida fit | 3.00 | 3.69 | 21.00 | 0.50 | 1 |

Supplementary Table 1: Specifications of Scanners and Scanning Parameters used for TOF MRIs for ID.

|  | **Main Institute** | **Affiliate Center 1** | **Affiliate Center 2** | **Affiliate Center 3** |
| --- | --- | --- | --- | --- |
| **% of Institutional Data** | 53% | 41% | 3% | 2% |

Supplementary Table 2: Sources of TOF-MRIs for Institutional Data

Figures


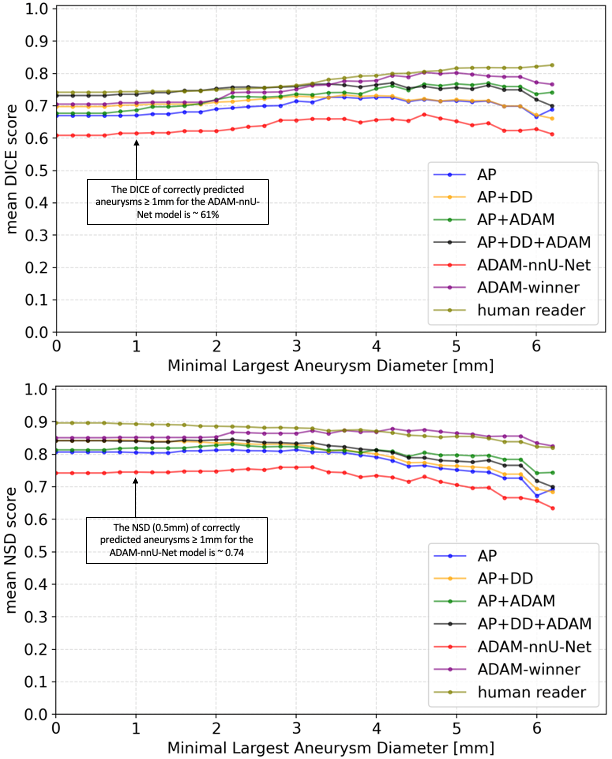


Supplementary Figure 1: Diameter-dependent DICE and NSD scores for all models. The figure shows mean DICE and NSD scores for all correctly detected UICA that are equal or larger than the diameter specified on the x-axis.
